# Comprehensive Analysis of a Ferroptosis-Related lncRNA Signature for Predicting Prognosis and Immune Landscape in Osteosarcoma

**DOI:** 10.3389/fonc.2022.880459

**Published:** 2022-06-28

**Authors:** Yiming Zhang, Rong He, Xuan Lei, Lianghao Mao, Zhengyu Yin, Xinyu Zhong, Wenbing Cao, Qiping Zheng, Dapeng Li

**Affiliations:** ^1^ Department of Orthopedics, Affiliated Hospital of Jiangsu University, Zhenjiang, China; ^2^ Cancer Institute, The Affiliated People’s Hospital of Jiangsu University, Zhenjiang, China; ^3^ Department of Burn and Plastic Surgery, Affiliated Hospital of Jiangsu University, Zhenjiang, China; ^4^ Department of Hematological Laboratory Science, Jiangsu Key Laboratory of Medical Science and Laboratory Medicine, School of Medicine, Jiangsu University, Zhenjiang, China; ^5^ Shenzhen Academy of Peptide Targeting Technology at Pingshan, and Shenzhen Tyercan Bio-Pharm Co., Ltd., Shenzhen, China

**Keywords:** osteosarcoma, ferroptosis, lncRNA, immune microenvironment, prognosis

## Abstract

Research on the implications of ferroptosis in tumors has increased rapidly in the last decades. There are evidences that ferroptosis is involved in several aspects of cancer biology, including tumor progression, metastasis, immunomodulation, and therapeutic response. Nonetheless, the interaction between ferroptosis-related lncRNAs (FRLs) and the osteosarcoma immune microenvironment is poorly understood. In this study, a risk model composed of FRLs was developed using univariate and LASSO Cox regression analyses. On the basis of this model, FRL scores were calculated to systematically explore the role of the model in predicting the prognosis and immune characteristics of osteosarcoma patients. Survival analysis showed that osteosarcoma samples with lower FRL-score had better overall survival. After predicting the abundance of immune cells in osteosarcoma microenvironment by single-sample gene-set enrichment analysis (ssGSEA) and ESTIMATE analysis, we found that the FRL-score could distinguish immune function, immune score, stromal score, tumor purity, and tumor infiltration of immune cells in different osteosarcoma patients. In addition, FRL-score was also associated with immune checkpoint gene expression and half-maximal inhibitory concentration of chemotherapeutic agents. Finally, we confirmed that knockdown of RPARP-AS1 suppressed the malignant activity of osteosarcoma cells *in vitro* experiments. In general, the FRL-based prognostic signature could promote our understanding of the immune microenvironment characteristics of osteosarcoma and guide more effective treatment regimens.

## Introduction

Osteosarcoma is the most common primary malignant bone tumor, often occurring in the epiphyses of the extremities, especially the proximal tibia and distal femur (1). It has been currently the second leading cause of tumor-related death in adolescents due to the inferior prognosis, frequency of recurrence, and distant metastasis (2–4). Standard treatment with preoperative chemotherapy, surgery, and postoperative adjuvant chemotherapy has resulted in a 5-year survival rate of 60%-70% for patients with localized osteosarcoma (5–7). However, the early clinical signs of osteosarcoma are not apparent and specific (8). More than one-fifth of osteosarcoma patients have pulmonary metastases at the time of diagnosis, leading to ineffective current treatment options and a dramatic decrease in survival to only 15-20% for such patients (9–11). Not only that, the present conventional treatments such as radical surgery and systemic chemotherapy are also associated with adverse effects such as trauma, liver dysfunction, and cardiotoxicity, which seriously impact the quality of life for patients (12–15). In addition, osteosarcoma is a highly heterogeneous tumor that often exhibits abnormal gene regulation and epigenetic mechanisms (16, 17). Many molecularly targeted drugs are only effective in some patients, which poses a considerable challenge to treating osteosarcoma (18–20). Therefore, it is imperative to explore reliable and effective prognostic biomarkers and therapeutic targets to guide clinical decision-making and personalize the treatment of osteosarcoma.

LncRNAs are a class of non-coding RNAs with transcript lengths over 200 nt, which are involved in cell cycle regulation, DNA damage repair, proliferation, differentiation, and other biological processes (21, 22). With the continuous development of epigenomics and large-scale transcriptomic studies, the role of lncRNAs in osteosarcoma pathogenesis, malignant biological behavior, therapeutic efficacy, and prognosis has been revealed (23). For example, Pan et al. found that lncRNA HCG18 promotes aerobic glycolysis and proliferation of osteosarcoma *in vitro* and *in vivo* through the miR-365a-3p/PGK1 axis (24). Ding et al. also have indicated that lncRNA MELTF-AS1 is associated with poor patient prognosis, and it can regulate MMP14 expression *via* competitively binding to miR-485-5p, thereby enhancing the metastatic ability of osteosarcoma cells (25). Another study showed that LAMTOR5-AS1 is involved in regulating proliferation, apoptosis, and chemotherapy-induced cellular oxidative stress in osteosarcoma cells by hindering the nuclear localization of NRF2 (26). In conclusion, these studies suggest that lncRNAs could be used as potential molecular markers and therapeutic targets with promising clinical applications in the early diagnosis and treatment of osteosarcoma.

Ferroptosis is a recently identified form of programmed cell death characterized by increased intracellular free iron and lipid peroxide accumulation (27, 28). Unlike pyroptosis and autophagy, ferroptosis is iron-dependent and regulates cell death through the lethal accumulation of lipid peroxidation (29).The classical regulation of ferroptosis relies on the neutralization of lipid peroxides by the glutathione peroxidase 4 (GPX-4) (30–32). There is growing evidence that cancer cells of higher malignancy, especially in intrinsically or acquired drug-resistant tumor cells, show hypersensitive to ferroptosis (33, 34). Notably, the classical chemotherapeutic agent for osteosarcoma, cisplatin, has been shown to induce ferroptosis in cancer cells, with significant synergistic effects with other ferroptosis inducers (35, 36). These studies undoubtedly bring new directions for the treatment of drug-resistant osteosarcoma. Moreover, ferroptosis is associated with T cell-mediated antitumor immunity and affects the efficacy of cancer immunotherapy (37). It has also been shown that immunomodulation of the tumor microenvironment (TME) can promote ferroptosis, which in turn enhances the response to immunomodulation by strengthening the immunogenicity of the TME (38). The combination of immunotherapy with modalities that promote ferroptosis, such as radiation therapy and targeted therapy, is expected to produce synergistic effects through ferroptosis to promote tumor control (39, 40). LncRNAs have been shown to be associated with the biological process of ferroptosis, thereby affecting cancer growth. It has been shown that lncRNAs can act as regulators of ferroptosis by regulating GPX4 activity, Fe 2+ levels, cysteine metabolism, etc. in ferroptosis (41). GPX4 is a GSH-dependent enzyme whose antioxidant function requires the use of GSH as a substrate, thereby reducing lipid peroxidation and inhibiting ferroptosis (42, 43). Ketamine induces cell growth inhibition and ferroptosis by targeting the lncRNA PVT1/miR-214-3p/GPX4 axis (44). Overexpression of lncRNA NEAT1 suppressed the expression of ACSL4, while increasing the expression of SLC7A11 and GPX4, thereby reducing the occurrence of ferroptosis and apoptosis (45). Fenton reaction activation and subsequent elevation of ROS levels lead to lipid peroxidation in ferroptosis arising from increased intracellular Fe ^2+^ (46). LncRNA PVT1 regulated iron levels and ferroptosis in cortical peri-infarct region of ischemia/reperfusion mice (47). Excessive depletion of intracellular GSH can inhibit GPX4 from exerting its antioxidant effects, leading to ferroptosis (48). Knockdown of lncRNA MEG3 reduces P53 and promotes GSH synthesis, leading to lower ROS levels (49). It has also been shown that LncRNA P53RRA regulates metabolic genes at the transcriptional level to promote ferroptosis and apoptosis (50). However, the role of ferroptosis-related lncRNAs (FRLs) in the clinical prognosis and immunotherapy of osteosarcoma has been rarely reported. Therefore, an in-depth study of ferroptosis and tumor immune-related biomarkers is essential to improve the immunotherapeutic outcome of osteosarcoma.

The construction of prognostic models through bioinformatics approaches can help explore the clinical significance of lncRNAs and optimize decision making for individual tumor patients. An immune-derived lncRNA signature, as developed by machine learning-based integration, helps to assess the prognosis of fluorouracil-based adjuvant chemotherapy and the benefit of immune checkpoint inhibitors (ICI) therapy (51). A consensus machine learning-derived lncRNA signature identified by a combination of 76 algorithms was able to efficiently identify patients at high risk of relapse and help predict the benefit of bevacizumab therapy in patients with stage II/III colorectal cancer (52). In this study, we screened prognosis-related FRLs and stratified osteosarcoma patients into two subtypes with different prognoses based on the expression levels of these lncRNAs. Subsequently, an FRLs-based signature was constructed to predict the outcome of individual patients and guide clinical decision-making. We validated the clinical application value of the signature and discussed its correlation with different clinicopathological factors. Further analysis shows that the FRLs-based signature could also predict the characteristics of the tumor immune microenvironment (TIME), response to chemotherapy, and immunotherapy in osteosarcoma. Finally, we knocked down the expression of the critical lncRNA RPARP-AS1 to detect the effect on the proliferation and migration of osteosarcoma cells *in vitro*. Our results suggest that Ferroptosis-related lncRNAs may play an essential role in osteosarcoma prognosis and immunotherapy.

## Materials and Methods

### Data Source and Differential Expression of Ferroptosis-Related Genes

The RNA sequencing (RNA-seq) data for normal muscle tissue samples were downloaded from the GTEx database (https://www.gtexportal.org/home/). Gene expression data and corresponding clinical information from osteosarcoma samples were downloaded from TARGET (https://ocg.cancer.gov/programs/target). A total of 484 samples were obtained, including 88 osteosarcoma samples and 396 normal samples (tumor samples were all from the TARGET database, and normal samples were all from the GTEx database) (53, 54). The lncRNA and mRNA expression profiles were extracted from the expression data of 88 patients with osteosarcoma, respectively. All gene expression levels were normalized using log2 (FPKM+1). The list of 259 ferroptosis-related genes (FRGs) from FerrDb (http://zhounan.org/ferrdb) is shown in **Supplementary Table S1** (55). We used the “limma” package to identify differentially expressed ferroptosis-related genes (DEFRGs) by a |log2FC| > 0 and false discovery rate (FDR) < 0.05 (56). The “clusterProfiler” package was used to perform GO and KEGG enrichment analysis on FRGs. All DEFRGs were imported into the STRING database (https://string-db.org/), a comprehensive tool for gene annotation and functional analysis to construct the protein−protein interaction (PPI) network. We set the PPI screening criterion as a minimum required interaction score > 0.4. Then, we used Cytoscape (http://www.cytoscape.org/) for visual analysis of the network.

### Identification of Ferroptosis-Related LncRNAs and Consensus Clustering Analysis

Pearson correlation analysis was performed based on the differential expression FRGs to obtain FRLs under the screening criteria of correlation coefficient >0.5 and P-value<0.001. We then performed univariate Cox regression analysis with the “survival” package to identify FRLs significantly associated with prognosis. Wilcoxon test was used to detect differences in expression of prognosis-related FRLs between tumor tissues and normal tissues. The correlation between PD-L1 expression and these FRLs was calculated by the “limma” and “corrplot” package. Subsequently, we used the “ConsensusClusterPlus” package for unsupervised consensus clustering to identify potential subtypes of FRLs. The clustering index “k” was increased from 2 to 10 to determine the optimal number of clusters for increasing intra-group correlation and decreasing inter-group correlation after clustering. For survival analysis of the subgroups, Kaplan-Meier survival curves were plotted using the “survival” and “survminer” packages. The differences in age, metastatic status, and gender between subtypes were further compared to examine the clinical value of the FRLs molecular subtypes. We also estimated the relative proportions of 22 different immune cell subpopulations between the two subtypes using the “CIBERSORT” package.

### Construction and Validation of Ferroptosis-Related LncRNA Signature

We then constructed an FRL prognostic signature to quantify ferroptosis patterns in individual osteosarcoma patients. Based on prognosis-related FRLs screened by univariate Cox regression, we performed Lasso regression analysis to avoid overfitting and construct the best prognostic signature. The FRL prognostic signature score, which we named “FRL-score,” was calculated as follows: FRL-score=Σ (βi × Expi) (β: coefficients, Exp: lncRNA expression level). Since few datasets are containing FRLs in osteosarcoma, we randomly divided the osteosarcoma patients with survival data in the TARGET database into a test set (41) and a training set (44) using the “caret” package (57). We constructed an FRL prognostic signature in the training set and validated it using the test set and the whole set. Kaplan-Meier survival analysis was performed by “survivor” and “survminer” package to compare the OS time of patients in different FRL-score subgroups. Time-dependent receiver operating characteristic (ROC) analysis of overall survival was used to assess the sensitivity and accuracy of the prognostic model by the “survivalROC” package. For survival analysis, a P-value <0.05 and an area under ROC (AUC) >0.70 was considered an acceptable predictive value. Finally, the principal component analysis (PCA) of all osteosarcoma samples was performed by the “limma” and “scatterplot3d” R packages.

### Comparison of FRL Prognostic Signature With Different LncRNA Prognostic Signatures and Clinical Features

We obtained relevant data from recent literature on lncRNA-based prognostic signatures for osteosarcoma, such as: metabolism-related nine-lncRNA signature (58), metabolism-related eight-lncRNA signature (59), and pyroptosis-related three-lncRNA signature (60). We named these three signatures as Chen signature, Gong signature, and Bu signature according to the names of the authors, respectively. We compared these signatures with our FRL signature by ROC analysis using the “limma”, “Survival”, “survminer”, and “timeROC”, packages. Metastasis is considered a major factor affecting the prognosis of patients with osteosarcoma (45–48). More than one-fifth of osteosarcoma patients have pulmonary metastases at the time of diagnosis, with survival rates for this group of patients dropping dramatically to only 15-20% (9–11). We therefore tried to compare FRL signature with metastasis and other clinical features based on clinical data from the TARGET database for patients with osteosarcoma by performing ROC analysis using the “Survival”, “survminer”, and “timeROC”, packages.

### Independent Prognostic Analysis and Clinical Utility of the FRL Prognostic Signature

We further assessed whether the FRL-score was independent of other traditional clinical characteristics using univariate and multivariate Cox regression analyses. Patients with osteosarcoma were subsequently divided into two subgroups according to age (≤18 and > 18 years), gender (female and male), and metastatic stage (M0 and M1) (54). Stratified analysis was performed to evaluate further the predictive ability of the FRL-score in each subgroup. In addition, we also assessed the correlation between the FRL-scores, immune scores, molecular subtypes, age, sex, and metastatic status using the “limma” and “ggpubr” package. Finally, we created a nomogram including the FRL-score and different clinical characteristics to predict the OS of osteosarcoma patients at 1, 3, and 5 years. We assessed the accuracy of the nomogram with a calibration chart using the “regplot”, “survival”, and “rms” package.

### Gene Set Enrichment Analysis of the Subgroups

The c2.cp.kegg.v7.4.symbols.gmt and c5.go.v7.4.symbols.gmt were downloaded from the MSigDB database (61). Based on the two reference gene sets described above, gene set enrichment analysis (GSEA) for subgroups was performed *via* the “limma”, “org.Hs.eg.db”, “clusterProfiler”, and “enrichplot” packages, bounded by P<0.05.

### Analysis of Tumor Immune Microenvironment, Drug Sensitivity and Response to Immunotherapy

We performed the ssGSEA and ESTIMATE algorithms to better appraise differences in the immune microenvironment characteristics of osteosarcoma between different FRL-score subgroups. The ESTIMATE algorithm is a method to infer the ratio of mesenchymal and immune cells in tumor samples using gene expression characteristics, i.e., to estimate the content of mesenchymal and immune cells in malignant tissues using expression data, to predict the immune score, stromal score, and tumor purity of each tumor sample (62). Immune scores, stromal scores, and tumor purity for each osteosarcoma patient were obtained using the “estimate” package. Single-sample Gene Set Enrichment Analysis (ssGSEA) was performed to assess the extent of immune cell infiltration and immune function in osteosarcoma, using the “GSVA” package. ssGSEA is a popular enrichment algorithm widely used in medical research, which is used to quantify the relative abundance of each cell infiltrate in osteosarcoma TME (63, 64). We then evaluated the value of the FRL-score in predicting drug sensitivity to common chemotherapeutic and molecularly targeted agents by analyzing half-maximal inhibitory concentrations (IC50) using the “pRRophetic” package. Finally, we examined the differential expression levels of the immune checkpoint genes between high and low FRL-score subgroups to predict the response of osteosarcoma patients to ICI using the “limma”, “reshape2”, “ggplot2”, and “ggpubr” packages.

### Cell Culture and Transfection

The human osteoblast cell line (hFOB1.19) and osteosarcoma cell lines (MG-63, MNNG/HOS, and U-20S) were obtained through the Cell Bank of the Chinese Academy of Sciences (Shanghai, China). MG-63 and MNNG/HOS cells were cultured in DMEM (BI, USA) mixed with 10% fetal bovine serum (FBS, BI, USA) and 1% penicillin/streptomycin. The hFOB1.19 cells were cultured in DMEM/F12 medium (BI, USA) containing 10% FBS and 1% penicillin/streptomycin, while U2OS cells were maintained in McCoy’s 5A medium. We cultured the cells in a humidified environment at 37°C and 5% CO2. Si-RPARP-AS1 and si-NC were obtained from Gemma Gene (Suzhou, China), and the sequence is shown in **Supplementary Table S2**. Osteosarcoma cells were freshly planted at 70–80% confluence and then transfected with Lipofectamine 3000 (Invitrogen, CA, USA) according to the instructions.

### Quantitative Real-Time PCR (qRT-PCR)

Total RNA from osteosarcoma cell lines and hFOB1.19 cells was extracted with RNA-easyTM Isolation Reagent (Vazyme Biotech Co., Ltd, Nanjing, China) and reversely transcribed into cDNA using a PrimerScript reagent kit (Takara, Beijing, China). QRT-PCR was conducted using UltraSYBR Mixture Kit (CWBIO, China). The comparative 2−ΔΔCt method was applied to determine relative expression, and GAPDH was chosen as the internal reference. Primer sequences for relevant genes are provided in **Supplementary Table S2**.

### Cell Proliferation, Invasion, and Migration Assays

Osteosarcoma cells were plated into a 96-well plate at a density of 3 × 10^3^ cells per well. Then ten μL Cell Counting Kit-8 (CCK-8) reagent (Yeasen Biotech, Shanghai, China) was added to each well according to the manufacturer’s protocol at 0, 24, 48, and 72 h. After incubation for two hours, the plates were detected using a microplate reader at 450 nm. Colony formation was also used to evaluate the cell proliferation activity. Osteosarcoma cells were seeded into 6-well plates with 500 cells/well and incubated with 37°C, 5% CO2 for 14 days. Then cells were fixed with 4% paraformaldehyde and stained with crystal violet. For transwell assay, osteosarcoma cells were seeded into the upper transwell chamber with 5 × 10^4^ cells/well and incubated with FBS-free medium at 37°C. For invasion, cells were seeded into the upper chamber with precoated Matrigel (Corning Life Science, MA, USA). The bottom transwell chamber was supplied 500 uL medium with 10% FBS. After 24 h, the cells that had migrated or invaded to the lower surface were fixed with 4% paraformaldehyde and stained with crystal violet for 15 minutes.

### Statistical Analysis

All statistical analyses were performed with R software (v4.0.5). We expressed measurement results as mean ± standard deviation (SD). We performed Student’s t-tests or one-way ANOVA to determine differences between groups. Statistical significance is indicated by *p < 0.05 and **p < 0.01.

## Results

### Screening of FRGs in Osteosarcoma

**Figure 1:**
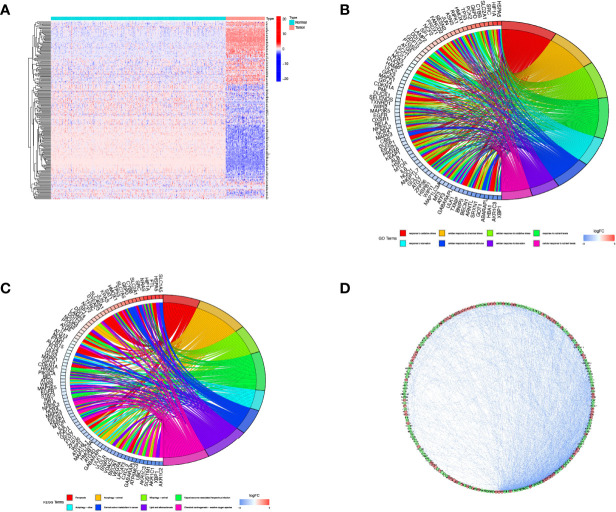
Screening of DEFRGs between osteosarcoma and normal tissues. **(A)** Heatmap for 227 DEFRGs. DEFRGs with upregulation, downregulation, and no significant difference were indicated by red, blue, and white dots, respectively. **(B)** GO enrichment analysis of DEFRGs. **(C)** KEGG pathway enrichment analysis of DEFRGs. **(D)** Protein-protein interaction network of DEFRGs. DEFRGs, differentially expressed ferroptosis-related genes; GO, Gene Ontology; Kyoto Encyclopedia of Genes and Genomes.

We compared the expression levels of 259 FRGs in the human osteosarcoma samples and normal muscle tissues. Based on our established cut-off standards, 227 DEFRGs were identified and used in the subsequent study (**Figure 1A**). We then performed GO annotation and KEGG pathway enrichment analysis to explore the potential biological functions of these DEFRGs and their relevance to ferroptosis. Go enrichment results showed that the DEFRGs were mainly enriched in response to oxidative stress, cellular response to chemical stress, cellular response to oxidative stress, and response to nutrient levels (**Figure 1B**). For KEGG enrichment, the DEFRGs were significantly enriched in ferroptosis, autophagy-animal, mitophagy-animal, and HIF-1 signaling pathways (**Figure 1C**). Moreover, the PPI network was constructed to predict protein-protein interactions between DEFRGs, which includes 207 nodes and 2076 edges (**Figure 1D**).

### Identification of Prognosis-Related FRLs in Osteosarcoma

**Figure 2:**
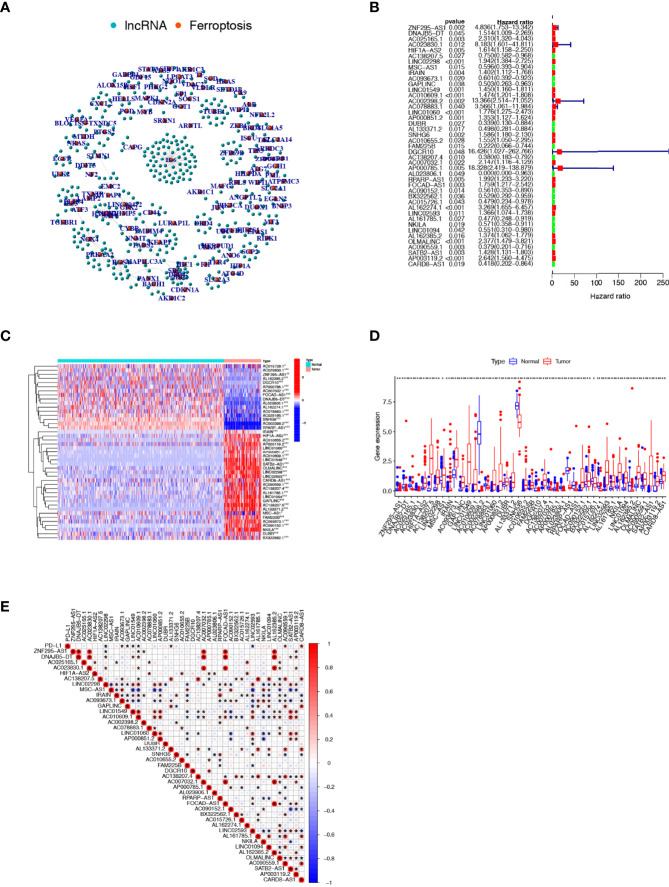
Comprehensive analysis of prognosis-related FRLs in osteosarcoma**. (A)** Co-expression network of FRGs and FRLs. **(B)** Identification of candidate prognosis-associated FRLs using univariate Cox regression analysis. **(C, D)** Heatmap **(C)** and boxplot **(D)** showing the expression level of prognosis-related FRLs. **(E)** correlation between prognosis-related FRLs and PD-L1 expression. FRGs, ferroptosis-related genes; FRLs, ferroptosis-related lncRNAs. *p < 0.05, **p < 0.01, *p < 0.001.

Based on Pearson correlation analysis, we identified 409 FRLs under the screening criteria of correlation coefficient >0.5 and P-value<0.001 (**Figure 2A**). To fully understand the impact of FRLs on the survival of osteosarcoma patients, we performed univariate Cox regression analysis and identified 43 prognosis-associated FRLs (**Figure 2B**), of which 17 lncRNAs were identified as protective factors with a hazard ratio (HR) <1, while the other 26 lncRNAs were considered as risk factors. We also examined the expression levels of these prognosis-related FRLs. As shown in **Figures 2C, D**, all 43 FRLs were differentially expressed between osteosarcoma samples and control tissue samples, with 27 FRLs upregulated versus 16 FRLs downregulated in osteosarcoma samples. In addition, we explored the correlation between the expression levels of prognosis-related FRLs and the immune checkpoint gene PD-L1. Our investigation revealed that AC093673.1, GAPLINC, AL133371.2, AC090559.1, and CARD8-AS1 were positively correlated with the expression levels of PD-L1, while LINC02298, LINC01549, AC010609.1, and LINC02593 were negatively correlated with the expression level of PD-L1 ( **Figure 2E** **).**

### Molecular Subgroups of Osteosarcoma Based on FRLs

**Figure 3:**
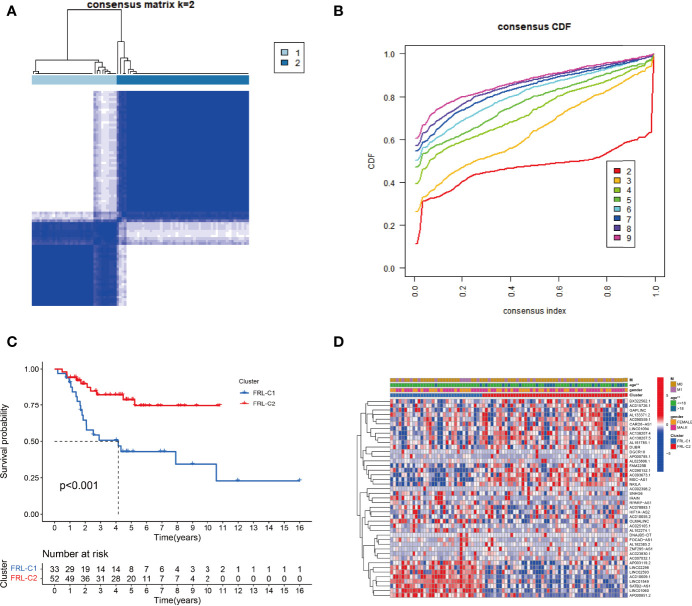
The construction of FRL-based osteosarcoma subtypes. **(A)** Consensus clustering matrix for k = 2. **(B)** The cumulative distribution function curve for K = 2–10. **(C)** The survival analysis of two molecular subtypes. **(D)** Heatmap showing the differential expression of prognosis-related FRLs between two subgroups. FRLs, ferroptosis-related lncRNAs. **p < 0.01.

To explore the expression profile of FRLs in osteosarcoma, we subsequently segmented osteosarcoma patients according to the expression of these FRLs by consensus clustering. Our results showed that k=2 was the optimal number of clusters with the highest intra-group correlation and the least inter-group interference (**Figures 3A, B**). Therefore, osteosarcoma patients were divided into two clusters named FRL-C1 and FRL-C2. The survival analysis results showed a significant survival advantage for FRL-C2 patients (**Figure 3C**). The heatmap shows the differential expression of prognosis-related FRLs between the different clusters, with FRL-C1 characterized by AP003119.2, LINC02298, LINC02593, AC010609.1, LINC01549, SATB2-AS1, LINC01060, and AP000851.2 with increased expression, and FRL-C2 showing high expression of FAM225B, AC090152.1, AC093673.1, MSC-AS1, and NKILA (**Figure 3D**). In addition, we noted a significant difference in age between the two clusters but little difference in other clinicopathological features (**Figure 3D**).

### Construction and Validation of the FRL-Based Prognostic Signature

**Figure 4:**
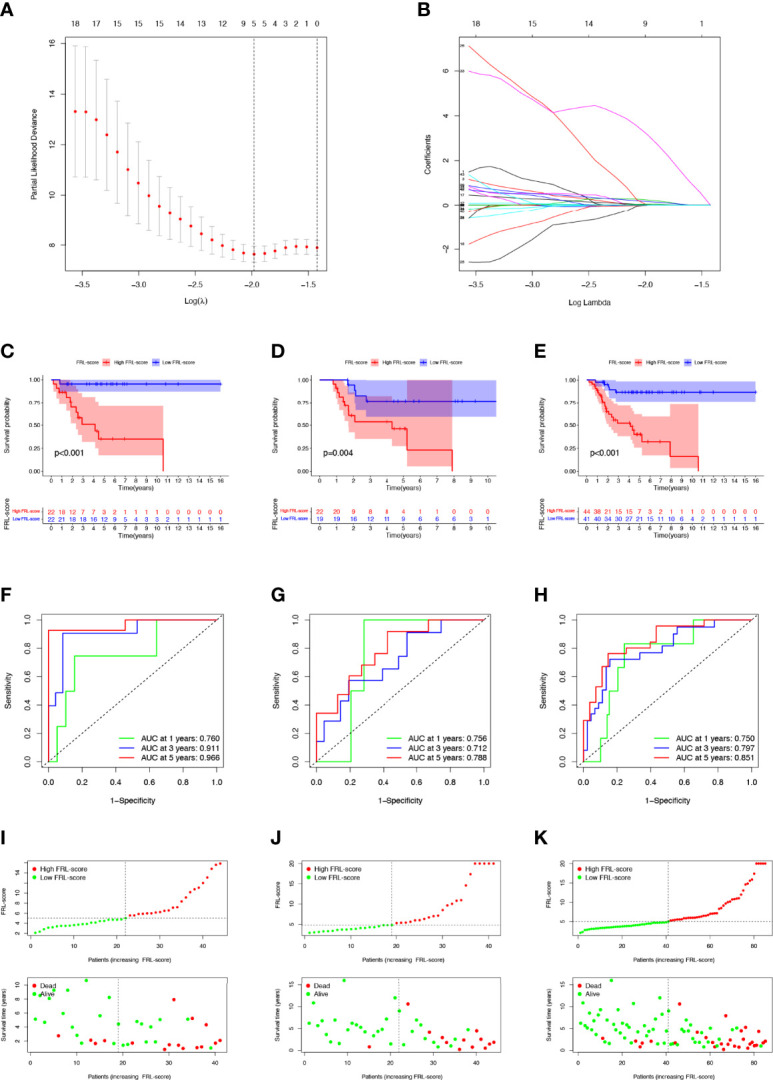
Construction and evaluation of the FRL-based prognostic signature. **(A, B)** Construction of the FRL-based signature by LASSO regression analysis. **(A)** Obtainment of the optimal λ value. **(B)** LASSO coefficient profiles of FRLs. **(C–E)** Survival analysis according to FRL-score in the training cohort **(C)**, validation cohort **(D)**, and whole cohort **(E)**. **(F–H)** ROC curve for forecasting overall survival in the training cohort **(F)**, validation cohort **(G)**, and whole cohort **(H)**. **(I–K)** The distribution of FRL-score, survival status, and survival time of patients in the training cohort **(I)**, validation cohort **(J)**, and whole cohort **(K)**. FRLs, ferroptosis-related lncRNAs.

To more accurately guide the treatment strategy and predict the prognosis of individual osteosarcoma patients, we next constructed a prognostic model of FRLs. We performed LASSO regression analysis on 43 prognosis-related FRLs screened by univariate Cox regression analysis and finally identified 5 FRLs for constructing the prognostic model (**Figures 4A, B**). The FRL-score formula is as follows: FRL-score = [LINC02298 expression × (0.241)] + [AP000851.2 expression × (0.026)] + [SNHG6 expression × (0.171)] + [RPARP-AS1 expression × (0.058)] + [AL162274.1 expression × (3.155)]. Patients were divided into low FRL-score and high FRL-score groups according to the median value of the FRL-score.

In the training set, patients with high FRL-score had significantly worse OS than those with low FRL-score, according to Kaplan-Meier survival analysis (**Figure 4C**). Similarly, in the validation set and the whole cohort, the prognosis of osteosarcoma patients in the high FRL-score group was significantly worse (**Figures 4D, E**). In the training cohort, the ROC analysis demonstrated the FRL-score in predicting survival of osteosarcoma patients was efficient, with AUC values of 0.760 (1 year), 0.911(3 years), and 0.966 (5 years), respectively (**Figure 4F**). ROC analyses in the validation set and the entire cohort also showed AUC values greater than 0.7 at 1, 3, and 5 years, again confirming the reliable prognostic performance of the FRL-score (**Figures 4G, H**). **Figures 4I–K** show the FRL-score, survival status, and survival time for patients of different subgroups in the training set, validation set, and the whole cohort. The results show that the mortality rate is higher in the high FRL-score subgroup.

**Figure 5:**
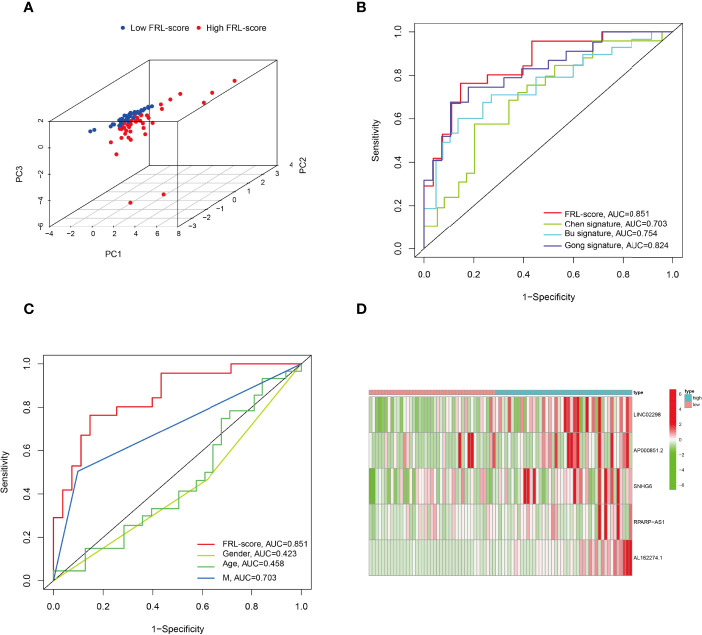
Principal component analysis and comparison of FRL prognostic signature with different prognostic signatures and clinical features. **(A)** Principal component analysis in the TARGET cohort. **(B)** Comparison of various lncRNA prognostic signatures in the TARGET cohort. **(C)** Comparison of FRL prognostic signature with different clinical features. **(D)** Expression heatmap of the five ferroptosis-related lncRNAs.

In addition, PCA analysis revealed that all patients in the TARGET cohort were well separated into two clusters depending on high and low FRL-score (**Figure 5A**). Thereafter, we compared the performance of different prognostic signatures and found that the AUC of FRL signature in the TARGET cohort was 0.851 for 5-year OS, which was significantly higher than Chen signature (AUC = 0.703), Bu signature (AUC = 0.754), and Gong signature (AUC = 0.824) (**Figure 5B**). As shown in **Figure 5C**, the AUC value of FRL signature for 5-year OS was 0.851, whereas the AUC value for metastasis was 0.703. This indicates the high accuracy of our prognostic model prediction relative to other clinical features such as metastasis. Finally, we showed the difference in the expression of five key FRLs between high and low FRL-score groups in all osteosarcoma patients with a heatmap (**Figure 5D**).

### Clinical Correlation Analysis and Stratified Analysis

**Figure 6:**
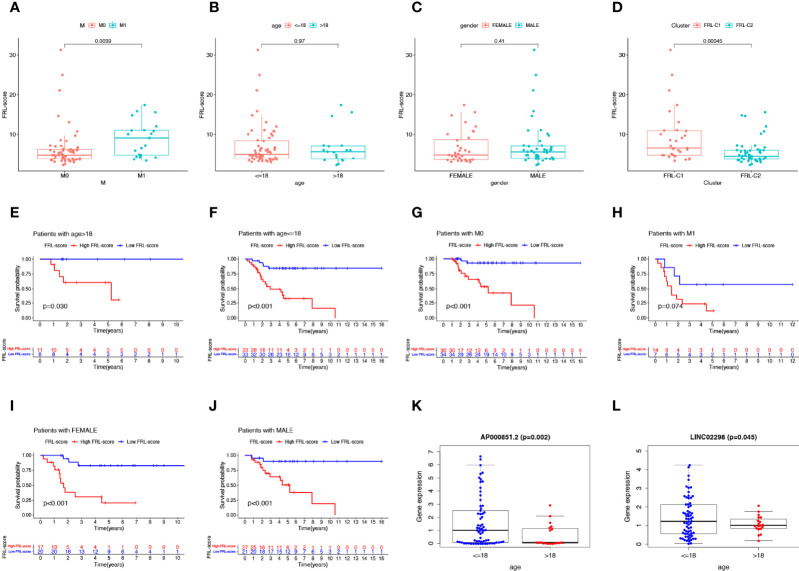
Clinical correlation analysis and stratification analysis of the FRL-score. **(A–D)** The correlation between the FRL-score and metastatic stage **(A)**, age **(B)**, gender **(C)**, and molecular subgroups **(D)**. **(E–J)** Stratification survival analysis of patients in different clinical subgroups, including women **(E)**, men **(F)**, M0 stage **(G)**, M1 stage **(H)**, age ≤65 **(I)**, and age >18 **(J)**. **(K)** Relationship between expression level of AP000851.2 and age category. **(L)** Relationship between expression level of LINC02298 and age category.

As shown in **Figures 6A–C**, we found that patients with osteosarcoma metastases had higher levels of FRL-score than patients without metastases, while there was no significant difference in FRL-score between patients of different ages and genders. In addition, we evaluated the FRL-score between the two molecular subtypes. Our results indicated that the FRL-score was higher in FRL-C1 than in FRL-C2 (**Figure 6D**). Based on the poorer prognosis of patients with high FRL scores, we hypothesized that the prognosis of FRL-C1 was worse. This result was consistent with the survival analysis of the molecular subtypes (**Figure 3C**). We then performed stratified survival analysis to further survey the predictive ability of FRL-score in multiple clinical subgroups, including gender (female or male), metastatic stage (M0 or M1), and age (≤18 or >18 years). In all other subgroups such as different sex subgroups, different age subgroups and M0 stage subgroup, patients with high FRL scores had worse OS than patients with low FRL scores, except for the M1 stage subgroup (**Figures 6E–J**). In addition, we found that the expression of two pivotal lncRNAs (AP000851.2 and LINC02298) also correlated with patient age (**Figures 6K, L**).

### Independent Prognostic Analysis and Nomogram Construction

**Figure 7:**
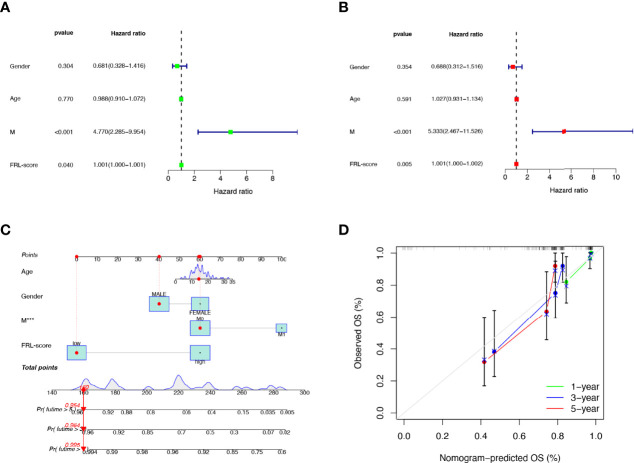
Prognostic value of different clinical parameters and construction of nomogram. **(A)** Univariate analysis of various clinical parameters and FRL-score in osteosarcoma patients. **(B)** Multivariate analysis of various clinical parameters and FRL-score in osteosarcoma patients. **(C)** Nomogram constructed using different clinical parameters and FRL-score for predicting OS in osteosarcoma patients. **(D)** Calibration plot of the nomogram for predicting OS. OS, overall survival.

To verify the independent prognostic value of the FRL-score under the influence of other clinical parameters, we performed univariate and multivariate Cox regression analyses. As shown in **Figures 7A, B**, FRL-score and metastasis could be used as independent prognostic indicators for patients with osteosarcoma. Subsequently, we created a nomogram containing the FRL-score and clinicopathological parameters to predict the OS of osteosarcoma patients at 1, 3, and 5 years (**Figure 7C**). We also plotted calibration curves at 1, 3, and 5 years to assess the accuracy of the nomogram (**Figure 7D**).

### GSEA of Different FRL-Score Subgroups

**Figure 8:**
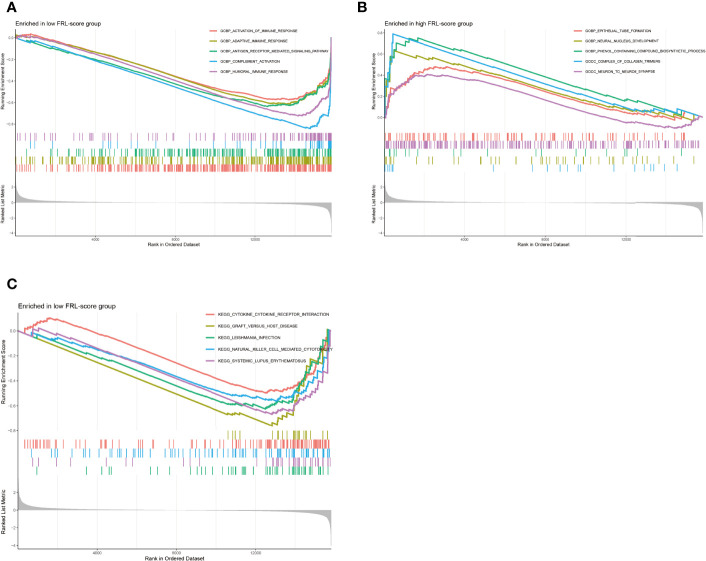
Gene set enrichment analysis of different FRL-score subgroups. **(A)** GO enrichment analysis of low FRL-score group. **(B)** GO enrichment analysis of high FRL-score group. **(C)** KEGG enrichment analysis of low FRL-score group.

We performed GSEA to assess potential differences in biological function between the FRL-score-classified subgroups. The GO functions enriched in the low FRL-score group were activation of immune response, adaptive immune response, antigen receptor mediated signaling pathway, complement activation, and humoral immune response (**Figure 8A**). In contrast, the high FRL-score group was mainly enriched in epithelial tube formation, neural nucleus development, phenol containing compound biosynthetic process, complex of collagen trimers, and neuron to neuron synapse (**Figure 8B**). Based on these results, we hypothesized that the better prognosis of patients with osteosarcoma in the low FRL-score group could be related to the activation of immunomodulatory functions. Similarly, the enrichment pathways in the low FRL-score group appear to be immune-related, such as natural killer cell mediated cytotoxicity, cytokine-cytokine receptor interaction, and graft versus host disease (**Figure 8C**). However, there were no enriched pathways in the high FRL-score group.

### Immune Microenvironment Characteristics Between Different FRL-Score Subgroups

**Figure 9:**
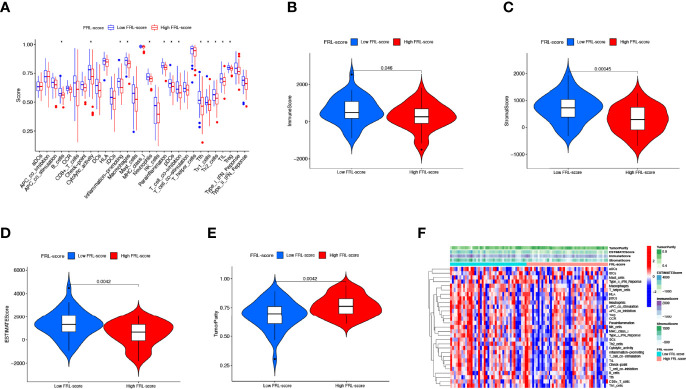
Immune characteristics of different FRL-score subgroups. **(A)** Disparities in immune cell infiltration and immune functions between different subgroups in the TARGET set. **(B)** Comparison of the immune scores between different FRL-score subgroups. **(C)** Comparison of the stromal scores between different FRL-score subgroups. **(D)** Comparison of the ESTIMATE scores between different FRL-score subgroups. **(E)** Comparison of tumor purity between different FRL-score subgroups. **(F)** Heatmap of the distinctions in immune microenvironment characteristics between the two subgroups.

Tumor development is implicated in the heterogeneity of the TME, such as infiltrating immune cells, extracellular matrix, tumor purity, and non-cellular components (65). Therefore, we explored the role of the FRL-score in predicting the immune microenvironment landscape of osteosarcoma. Our investigation revealed higher levels of tumor infiltration in the low FRL score group for B cells, macrophages, neutrophils, plasmacytoid dendritic cells, T follicular helper, tumor-infiltrating lymphocytes, regulatory T cells, and T helper cells, using the ssGSEA algorithm (**Figure 9A**). At the same time, the low-FRL subgroup had more active immune functions such as cytolytic activity, Inflammation−promoting, parainflammation, and T cell co-inhibition (**Figure 9A**). The ESTIMATE analysis showed that the immune score, stromal score, and ESTIMATE score were significantly lower in the high FRL-score group than in the low FRL-score group (**Figures 9B–D**). Consistent with the above results, the high FRL-score group had higher tumor purity (**Figure 9E**). The heatmap visualizes the distinctions in immune microenvironment characteristics between the two subgroups as derived from ssGSEA and ESTIMATE analyses (**Figure 9F**). Combined with the previous survival analysis results, we speculate that the heterogeneity of the TIME may be one of the reasons for the different prognoses of the high and low FRL-score groups.

### Drug Sensitivity Analysis and Immunotherapy Response Analysis

**Figure 10:**
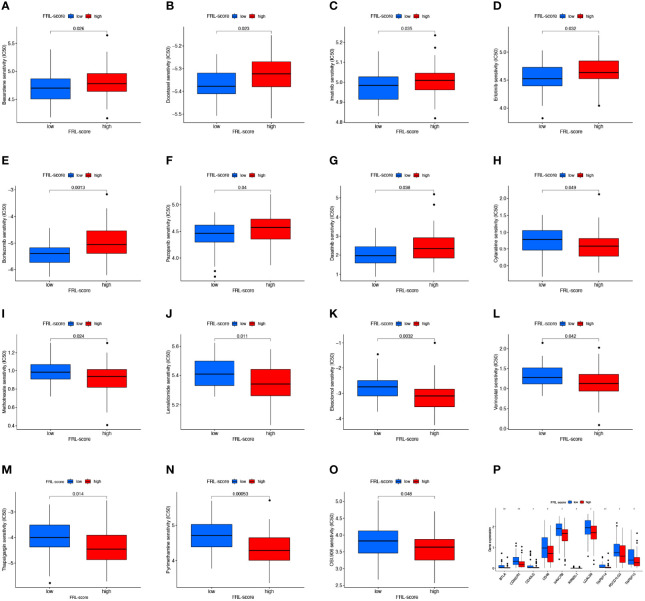
Drug sensitivity analysis and immune checkpoint molecules expression analysis. **(A–O)** Comparison of drug sensitivity between different FRL-score subgroups, including bexarotene **(A)**, docetaxel **(B)**, imatinib **(C)**, erlotinib **(D)**, bortezomib **(E)**, pazopanib **(F)**, dasatinib **(G)**, cytarabine **(H)**, methotrexate **(I)**, lenalidomide **(J)**, elesclomol **(K)**, vorinostat **(L)**, thapsigargin **(M)**, pyrimethamine **(N)**, and OSI-906 **(O)**. **(P)** Differential expression of immune checkpoint genes between different FRL-score subgroups.

To determine the relationship between FRL-score and anticancer drug sensitivity, we compared the difference in the half-maximal inhibitory concentration (IC50) of commonly used drugs between the FRL-score-classified subgroups. For the low FRL-score group, the IC50 values of bexarotene, docetaxel, imatinib, erlotinib, bortezomib, pazopanib, and dasatinib were lower, indicating that the low FRL-score patients were more sensitive to these seven drugs (**Figures 10A–G**). In contrast, cytarabine, methotrexate, lenalidomide, elesclomol, vorinostat, thapsigargin, pyrimethamine, and OSI-906 had lower IC50s in patients with high FRL-score. We next sought to predict the sensitivity of patients with different FRL scores to immune checkpoint inhibitor therapy (**Figures 10H–O**). As shown in **Figure 10P**, the expression of ten immune checkpoint genes was significantly altered between the two subgroups, with the high FRL-score group exhibiting lower levels of immune checkpoint gene expression. Our results demonstrate that the FRL-score can be used to predict immunotherapy response and chemotherapy drug sensitivity in patients with osteosarcoma, which can help guide clinical decision-making and individualized treatment regimens.

### Preliminary Functional Validation of RPARP-AS1

**Figure 11:**
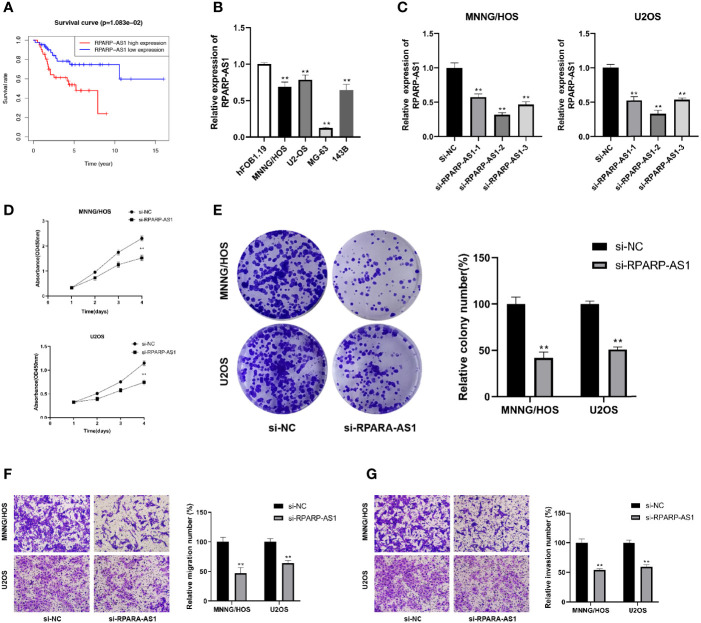
Downregulation of RPARP-AS1 inhibited osteosarcoma cell growth migration, and invasion. **(A)** Survival analysis of osteosarcoma patients with high and low RPARP-AS1 expression. **(B)** RPARP-AS1 expression in osteosarcoma cell lines and osteoblast cell line. **(C)** Transfection efficiency of si-RPARA-AS1 in MNNG/HOS and U2OS cells. **(D, E)** CCK-8 **(D)** and colony formation **(E)** assays were utilized to detect the proliferative capacity of MNNG/HOS and U2OS cells after RPARP-AS1 downregulation. **(F, G)** Transwell assays were applied to determine the migration **(F)** and invasion **(G)** capacity of MNNG/HOS and U2OS cells after RPARP-AS1 downregulation (magnification, 100×). **p < 0.01.

RPARP-AS1 has been documented to affect the prognosis of several solid tumors, such as triple-negative breast cancer, lung adenocarcinoma, and colon cancer (66–68). However, it is unclear whether RPARP-AS1 plays a specific role in osteosarcoma. Our previous analysis had identified RPARP-AS1 differentially expressed in osteosarcoma and control samples. Subsequent survival analysis also showed that high RPARP-AS1 expression was negatively correlated with prognosis in osteosarcoma (**Figure 11A**). We further examined the expression of RPARP-AS1 in osteosarcoma cell lines by qRT-PCR, which showed that RPARP-AS1 was lowly expressed in MG-63, MNNG/HOS, U2OS, and 143B cells (**Figure 11B**). The knockdown of RPARP-AS1 was performed in MNNG/HOS and U2OS cells to investigate its possible roles in osteosarcoma. As shown in **Figure 11C**, the knockdown effect of si-RPARP-AS1-2 was most pronounced and was selected for subsequent experiments. CCK-8 and colony formation assay suggested that inhibition of RPARP-AS1 dramatically suppressed cell viability in MNNG/HOS and U2OS cells (**Figures 11D, E**). Besides, knockdown of RPARP-AS1 suppressed MNNG/HOS and U2OS cells migration and invasion compared with the control group (**Figures 11F, G**).

## Discussion

Osteosarcoma is the most common primary bone malignancy worldwide (69). Although some progress has been made in treatment strategies such as radical surgery and neoadjuvant chemotherapy, the clinical prognosis of osteosarcoma is still unsatisfactory due to the rapid proliferative nature of tumor cells, high metastasis and chemotherapy resistance. Moreover, intra- and inter-tumor heterogeneity, as well as widespread genetic mutations, deletions, or amplifications in osteosarcoma, severely hinder the development of individualized patient treatment (70). To date, most patients with osteosarcoma have not received suitable targeted therapy tailored to their specific genomic alterations (71). Therefore, it is imperative to explore new therapeutic targets further and improve the survival of patients with osteosarcoma.

As a newly discovered type of cell death, ferroptosis is a hot topic of research, which occurs through lipid peroxidation, iron accumulation, reactive oxygen species production and glutathione deprivation, and has unique characteristics that show potential value in tumor research (72, 73). Most current therapeutic regimens work by inducing apoptosis in tumor cells, and once tumor cells undergo apoptotic escape, they become resistant to drugs, leading to reduced treatment sensitivity. As research progresses, the implications of ferroptosis in the development, progression, and multi-drug resistance of osteosarcoma are becoming better known, providing new avenues for treating osteosarcoma and overcoming chemoresistance (74). For illustration, it was found that inhibition of STAT3/Nrf2/GPX4 signaling induced ferroptosis sensitization, thereby enhancing the sensitivity of osteosarcoma cells to cisplatin (75). Lin et al. reported that EF24 could dose-dependently reduce GPX4 expression while releasing intracellular iron to promote ferroptosis in osteosarcoma by upregulating HMOX1 expression, which potentially serves as a therapeutic option for patients with HMOX1-positive osteosarcoma (76). Another study by Chen et al. demonstrated that KDM4A could inhibit ferroptosis by regulating the demethylation of the SLC7A11 promoter region. KDM4A expression was significantly upregulated in OS tumor tissues relative to normal tissues, while KDM4A knockdown promoted ferroptosis in osteosarcoma cells, limited pulmonary metastasis of osteosarcoma, and could act synergistically with cisplatin (77). MicroRNA-1287-5p promotes ferroptosis in osteosarcoma cells by inhibiting GPX4, but its expression is downregulated in human osteosarcoma relative to controls (78). These results suggest that ferroptosis may be regulated by different genes or non-coding RNAs in osteosarcoma, but the expression of these regulators differs in osteosarcoma and normal tissues.

Notably, lncRNAs are closely associated with ferroptosis in cancer by mediating the expression of lipid metabolism-related enzymes and ferroptosis-related genes at multiple levels, including RNA splicing and post-transcriptional (41). For example, the lncRNA GABPB1-AS1 has been implicated in erastin-induced ferroptosis, which could increase ROS production and negatively affect cellular antioxidant capacity, thereby inhibiting hepatocellular carcinoma cell growth (79). It has also been demonstrated that lncRNAs can regulate ferroptosis by interacting with p53 (47, 80). P53 has been a promising oncogenic molecule that induces apoptosis and cell cycle arrest, thus exerting powerful tumor-suppressive effects (81). Interestingly, p53 is also thought to promote the catabolism of fatty acids while inhibiting their synthesis. By regulating the expression of its metabolic targets such as GLS2, SLC7A11, PTGS2, SAT1, etc., p53 plays an essential role in regulating the ferroptosis response (82). According to Mao et al., lncRNA P53RRA could activate the p53 pathway in a dose-dependent manner by interacting with G3BP1 in the cytoplasm, thereby promoting ferroptosis in lung cancer (50). Zhang et al. showed that the binding of p53 to the lncRNA NEAT1 promoter increased the expression of lncRNA NEAT1 and served to promote ferroptosis in hepatocellular carcinoma cells (83). It is worth mentioning that hotspot mutations in p53 are usually immunogenic and elicit responses of intratumoral T cells to mutant p53 neoantigens, which have the potential to act as molecular targets in immunotherapy such as T cell receptor mimic monoclonal antibodies (81). Therefore, it would be interesting to investigate the contribution of FRLs and p53 in immunotherapy. Despite this, no studies have evaluated the role and possible mechanisms of lncRNA in the ferroptosis of osteosarcoma.

Here, we focused on the relationship between FRLs and the immune microenvironment, chemotherapy sensitivity, and prognosis of osteosarcoma. We identified new molecular subtypes of osteosarcoma, namely FRL-C1 and FRL-C2, based on 43 prognosis-related FRLs. The follow-up survival analysis and clinical correlation analysis demonstrated differences between subtypes, with FRL-C2 showing better survival outcomes and older age relative to FRL-C1. The above analysis was based on patient populations and demonstrated the overall effects mediated by FRLs in osteosarcoma. To allow stratification of each patient according to their genomic characteristics, we successfully constructed a prognostic model using five FRLs, including SNHG6, LINC02298, AP000851.2, RPARP-AS1, and AL162274.1. Numerous studies have confirmed the prognostic value and clinical potential of SNHG6 in cancer, serving as a competitive endogenous RNA as one of the primary mechanisms underlying its role (84). According to Xu et al., SNHG6 expression is abnormally upregulated and regulated by SP1 in colorectal cancer, not only that SNHG6 promotes epithelial-mesenchymal transition and accelerates colorectal cancer growth and metastasis by competitively bound to miR-26a/b and miR-214 (85). In addition, SNHG6 may also be involved in mRNA splicing or processing; Lan et al. demonstrated that SNHG6 increased the level of aerobic glycolysis and promoted proliferation in colorectal cancer by inducing hnRNPA1 to target PKM precisely (86). In our study, SNHG6 as a risk molecule was negatively associated with the outcome of osteosarcoma patients and was highly expressed in the high FRL-score group. In the study of Ruan et al., SNHG6 was relevant to the prognosis and pathological grading of osteosarcoma patients. It could drive cell cycle progression and promote osteosarcoma growth through the regulation of p21 and KLF2 (87). Another study also showed that SNHG6 inhibits autophagy and apoptosis but promotes metastasis in osteosarcoma *via* the miR-26a-5p/ULK1 axis (88). All of the above results indicate the oncogenic role of SNHG6 in osteosarcoma, which is consistent with our analysis. RPARP-AS1 promoted colon cancer proliferation and metastasis through competitive binding of mir-125a-5p while upregulating the expression of antiapoptotic-related markers (68). Notably, another study revealed that RPARP-AS1 was an N6-methyladenosine regulator in lung adenocarcinoma (LUAD) (67). Interestingly RPARP-AS1 is also an essential lncRNA associated with pyroptosis in osteosarcoma (60). Given the multiple effects of RPARP-AS1, we performed further analysis and preliminary experiments. The results suggested that high expression of RPARP-AS1 predicted decreased OS while downregulation of RPARP-AS1 inhibited osteosarcoma proliferation, invasion, and migration *in vitro*. Despite the low relative expression of RPARP-AS1 in osteosarcoma cell lines, survival analysis and preliminary experiments demonstrate that it may promote osteosarcoma progression. This observation seems paradoxical, but it makes sense. The role and expression of genes in osteosarcoma are influenced by multiple factors, such as tumor microenvironment, tumor heterogeneity, etc. Moreover, genes may perform numerous functions through different mechanisms, and it is not entirely reliable to infer the function of genes by relative expression. In addition, the specific roles and mechanisms of LINC02298, AP000851.2, and AL162274.1 in cancer have not been fully elucidated. The existing research revealed that LINC02298 might be involved in the upstream regulation of GPRIN1 and associated with the detrimental outcome of kidney renal papillary cell carcinoma and LUAD (89), while AP000851.2 may have an impact on stemness regulation in breast cancer (90). We fully validated the reliability of the FRL signature by ROC analysis, and comparison with other models or clinical features. These results further suggest that our constructed FRL signature have higher accuracy than previously reported lncRNA signatures and metastasis states. Because the FRL signature consists of only five lncRNAs, it has the advantage of being simple and inexpensive to calculate. Recent studies have demonstrated the detection of tumor-derived lncRNA in a variety of biological fluids including urine and blood (91–93). LncRNA has emerged an ideal non-invasive biomarker for cancer diagnosis and prognosis (94–96). Assays that distinguish patients from healthy controls by detecting lncRNA in serum or exosomes have simple and broad applicability (97, 98). However, our model still needs further validation in multiple clinical cohorts. In addition, exploring methods to detect model FRL in patient body fluids and reducing the cost of the assay is one of the future research directions. Furthermore, we have constructed nomograms based on the FRL-score and other clinical factors that allow accurate individual survival, which greatly improves the clinical utility of FRL signature in osteosarcoma patients with different characteristics. Therefore, patients can gain some insight into their condition based on their clinical features and FRL-scores, which may help in more accurate treatment.

The emergence of apoptotic escape and chemoresistance in osteosarcoma has been a concern. Immunotherapy is superior to conventional therapies and has been shown to benefit patients in the long term, making it a promising treatment for cancer (99). Tumors that attract more T-cell infiltration are reported to be “hot tumors,” which correspond to more sensitive immunotherapeutic effects (100). Therefore, we subsequently compared the degree of immune cell infiltration as well as the distribution of immune scores, stromal scores, and tumor purity in osteosarcoma patients with different FRL-score. We found the low FRL-score subgroup showed higher immune scores, stromal scores, immune cell infiltration, and immune functions. Consistent with the above results, the osteosarcoma patients with high FRL-score had high tumor purity. Unsurprisingly, our results also showed worse OS in the high FRL-score subgroup relative to the low FRL-score subgroup. We speculate suppression of both intrinsic and adaptive immunity may contribute to the worse prognosis among osteosarcoma patients in the high-scoring group. ICI is among the most well-studied drugs in the field of immunotherapy, and high immune checkpoint expression is one of the determinants for a favorable clinical response to ICI therapy (101, 102). Consistent with this observation, our results revealed that the immune checkpoint expression was higher in the low FRL-score subgroup. One explanation for their high OS rate may be that they could benefit from ICI treatment. Therefore, individualized treatment, based on FRL-score, is needed to select patients most likely to respond to ICI therapy. The above results confirm the role of the FRL-score in assessing the immune characteristic of osteosarcoma. In addition, the predicted results of chemotherapy drug sensitivity suggest ideal choices for diverse FRL-score groups of osteosarcoma patients. Different patients may be treated appropriately thus improving the response rate through the score.

Of course, there are some inevitable restrictions to our study. First, due to the inherent limitation of insufficient samples containing lncRNA expression profiles in clinical databases, further collection of osteosarcoma samples is necessary to verify the accuracy of this signature. At the same time, we recognize that we have only made preliminary explorations and that further experiments using animal models *in vivo* may better reveal the specific roles and mechanisms of the critical lncRNAs in osteosarcoma.

Overall, our study elucidates that FRLs could predict the outcomes of osteosarcoma patients and guide more personalized immunotherapy strategies by identifying the characteristics of TME.

## Data Availability Statement

The original contributions presented in the study are included in the article/**Supplementary Material** Further inquiries can be directed to the corresponding authors.

## Author Contributions

YZ and RH conceived of the research. XL and LM collected the data. YZ, RH, XL, XZ, ZY, and WC interpreted the data. YZ and RH drafted the manuscript. DL and QZ critically revised the manuscript. All authors read and approved the final manuscript.

## Funding

This work was supported by the Shenzhen Science and Technology Program (No.KQTD20170810154011370), the Youth Medical Key Talent Project of Jiangsu (QNRC2016844), the Natural Science Foundation of Jiangsu Province (BK20150475), “Six One Projects” for high-level health professionals in Jiangsu Province Top Talent Project (LGY2019089), medical education collaborative innovation Fund of Jiangsu University (JDY2022001), and Jiangsu Provincial key research and development program (BE2020679).

## Conflict of Interest

Author QZ was employed by the company Shenzhen Tyercan Bio-Pharm Co., Ltd.

The remaining authors declare that the research was conducted in the absence of any commercial or financial relationships that could be construed as a potential conflict of interest.

## Publisher’s Note

All claims expressed in this article are solely those of the authors and do not necessarily represent those of their affiliated organizations, or those of the publisher, the editors and the reviewers. Any product that may be evaluated in this article, or claim that may be made by its manufacturer, is not guaranteed or endorsed by the publisher.
