# Supplementary material for: Comprehensive Analysis of a Ferroptosis-Related lncRNA Signature for Predicting Prognosis and Immune Landscape in Osteosarcoma
Source: Front Oncol. 2022 Jun 28;12:880459. doi: 10.3389/fonc.2022.880459 (PMC9273977; doi:10.3389/fonc.2022.880459)
Supplement: Supplementary file 2 [file Table_2.docx]

**Supplementary Table S2. Primers for qRT-PCR and small interfering RNAs**

| Primers | | Sequence (5’-3’) |
| --- | --- | --- |
| RPARP-AS1 | F | GTCTGTCTACACCTGGGCTGGAG |
|  | R | GGGAGAATCGCTTGAACCTGGAAG |
| GAPDH | F | AAGCCCATCACCATCTTCCA |
|  | R | TAGACTCCACGACATACTCA |
| si-RPARP-AS1-1 | F | GCUAAUGGGCUGACCUUAATT |
|  | R | UUAAGGUCAGCCCAUUAGCTT |
| si-RPARP-AS1-2 | F | AAGAAAAGUAAAUAGGCCTT |
|  | R | GGCCUAUUUACUUUUCUUTT |
| si-RPARP-AS1-3 | F | UAAGAAAAGUAAAUAGGCTT |
|  | R | GCCUAUUUACUUUUCUUATT |
| si-NC | F | UUCUCCGAACGUGUCACGUTT |
|  | R | ACGUGACACGUUCGGAGAATT |
